# Comparison of the quality of clinical forensic examination of victims of physical violence conducted by clinicians and forensic examiners

**DOI:** 10.1007/s00414-023-02985-4

**Published:** 2023-03-25

**Authors:** Cleo Walz, Clara-Sophie Schwarz, Karla Imdahl, Christian Steffan, Tanja Germerott

**Affiliations:** 1 Institute of Forensic Medicine, Johannes Gutenberg University Medical Center, Am Pulverturm 3, 55131 Mainz, Germany; 2 Department of Criminology, Criminal Law and Medical Law, Johannes Gutenberg University, Mainz, Germany

**Keywords:** Victims of violence, Prosecution, Physical violence, Sexual violence, Istanbul Convention, Forensic standard

## Abstract

**Introduction:**

The Istanbul Convention calls for comprehensive care for victims of violence while maintaining forensic standards. After violent crimes, court usable documentation of injuries and securing of evidence is essential to avoid disadvantages for those affected in criminal prosecution.

**Material and methods:**

This retrospective study compares forensic relevant aspects in clinical forensic examination of victims of physical and sexual violence conducted by clinicians and forensic examiners. Forensic medical reports based on clinical documentation of individuals of all ages in the period from 2015 to 2018 (*n* = 132) were evaluated in comparison to a control group of examinations conducted by forensic specialists. A comparative statistical evaluation was performed.

**Results:**

The study revealed statistically significant differences in forensically relevant aspects. In the clinical examinations, full-body examination was performed in only 37.9%, and concealed body sites were examined in 9.8%. Photo documentation was often incomplete (62.4%), without scale (59.1%), blurred (39.7%), or poorly exposed (31.2%). Information on size, color, shape, and texture of injuries was often missing. In about every third examination, the findings were not described purely objective. A body scheme was used only in 8.3% of the clinical cases.

**Discussion:**

In order to establish nationwide care structures and the forensic standard required in criminal proceedings, intensive involvement of forensic medicine is essential. Standardized examination materials, regular training of medical staff, and telemedical approaches can improve the care for victims of violence regarding criminal prosecution.

## Introduction

Interpersonal violence is a global problem and has serious lifelong health and social consequences. Every year, 1.3 million people worldwide die as a result of violence of all kinds (self-directed, interpersonal, and collective), accounting for 2.5% of global mortality [1]. Nevertheless, deaths are just the tip of the iceberg. Women, children, and the elderly are most often affected by nonfatal physical, sexual, and psychological violence and neglect. In 2011, the Council of Europe therefore drew up the Convention on Preventing and Combating Violence against Women and Domestic Violence (Istanbul Convention) as an international treaty, which entered into force in 2014. The purpose of the Convention is to protect women from all forms of violence and to prevent, prosecute, and eliminate violence against women and domestic violence. According to Article 25 of the Convention, states are obliged to provide medical and forensic examinations after sexual violence, as well as trauma support and counseling. States parties are also required to maintain effective criminal justice standards and procedures for investigating and sanctioning acts of violence [2]. The implementation of these regulations is currently proceeding very heterogeneously in the individual countries. Even in Germany, there is not a comprehensive range of such facilities yet that meet the necessary medicolegal standards and are easily accessible for victims.

Frequently, victims of violence present themselves in a doctor’s office or clinic after experiencing violence. The acute forms and consequences of violence often require emergency care and are therefore presented in medical practices or emergency departments. Physical violence causes a wide variety of injury patterns, which are frequently named by the affected persons as consequences of accidents, which presents the treating physicians with corresponding challenges. Clinical forensic medicine is not an integral part of continuing medical education in Germany. In many cases, there is a lack of knowledge to identify injuries as consequences of violence. Physicians and other decision-makers often do not perceive violence as a public health problem; rather, violence is seen as a crime problem [1].

Physicians’ actions are generally geared to making a diagnosis and providing treatment as quickly as possible. In cases of physical and sexual violence, however, accurate and court usable injury documentation and trace evidence collection are of high importance for criminal proceedings. There, only findings that meet the forensic standard can be meaningfully interpreted by experts. In this way, the best possible starting situation for the persons affected is created, and at the same time, adverse decisions for the accused persons are avoided.

Availability of forensic evidence in cases of violence has been shown to benefit prosecution [3, 4]. From a legal point of view, the quality of medical examination and securing of evidence can be of vital importance in criminal proceedings for violent crime. This is mainly because the principle of “in dubio pro reo” (in doubt, for the accused) also applies in situations where the crime cannot be proven beyond reasonable doubt due to missing or inadequate medical documentation. These scenarios are most likely to occur in cases where the testimony of the person affected by violence is missing or remains as the only, but insufficient, evidence [5]. If, on the other hand, objective evidence based on medical documentation is available, there will be less need to rely on subjective testimony in court. In these cases, ambiguities based on one person’s testimony versus another’s are less likely to cause the criminal case to fail. In addition, the duration of proceedings can be considerably shortened if the findings are documented in accordance with the forensic standard.

In fields in which courts have no expertise of their own, they are dependent on professional expertise, to answer questions of law [6]. Medical evidence preservation is something that courts cannot do themselves. In any case, whether courts themselves or expert witnesses reconstruct a past event, recourse to preceding medical documentation is often indispensable.

From a legal perspective, the requirements for the quality of medical documentation derive from their relevance for a court’s decision-making in criminal proceedings. In trials, courts are supposed to determine the guilt of an alleged perpetrator and, if guilt is established, the legal consequences (for example, the amount of the penalty) of his or her action [7]. Guilt in this regard refers to whether a crime has been committed and if so, which one. For example, the fundamental distinction between guilt and innocence is affected, if it is not clear whether self-inflicted injury or third-party involvement has occurred. It can also make a big difference in the legal classification whether an assault was committed with or without a dangerous object or whether penetration occurred in a sexual offense. Apart from this, in cases of massive violence, the Federal Court of Justice in Germany not only assumes intent to commit assault but also regularly assumes intent to commit homicide [8]. Therefore, to determine the specific guilt and legal consequences, questions regularly arise in court about the presence and age of an injury, their origin and type, the force that caused it, and the circumstances under which the injury occurred, as well as consequential health damages [9]. Thus, anything that might provide evidence to answer these questions, or more precisely, anything that professionals (for example, forensic medical professionals) need to answer these questions, has legal relevance.

The present study examines the quality of medical documentation of findings and evidence collection regarding compliance with forensic standard. According to the standard DIN EN ISO 9000:2015–11 (valid standard for quality management), quality is defined as “the degree to which a set of inherent characteristics of an object fulfils requirements.” Quality refers to the degree of fulfillment of properties and characteristics of a product or service, i.e., the extent to which specified requirements are met [10]. Therefore, high-quality medical documentation can be said to exist if the completeness of all forensically relevant information is given, so that the injuries can be characterized in terms of their nature and a reconstruction of crime sequences is possible. The “Just_e_U!” project recommended a Europe-wide minimum standard for clinical forensic examinations [11].

The aim of the study is to compare the quality and identify deficits of clinical forensic examinations of victims of physical violence conducted by clinicians and forensic examiners.

## Material and method

In a retrospective cross-sectional study, cases of physical and sexual violence against persons of all ages (*n* = 132) were evaluated. These were cases in which a forensic medical report was drawn up on the basis of clinical documentation according to the files in the period from 01.01.2015 to 31.12.2018. Physical examinations performed directly by forensic medicine physicians (*n* = 132) were used as control group and matched to the case group based on the type of violence reported. In both groups, only those cases were included in which a criminal complaint was filed, and a forensic medical report was ordered before or after the examination. It should already be mentioned at this point that the present number of cases represents only a part of the reported cases, since forensic medical examinations or assessments based on medical records are not commissioned by the courts in all cases.

Based on the forensic medical case files, the data of the person examined, the type of violence, and the injuries and notes for substance impairment were evaluated. In addition, information on the circumstances of the examinations (time, place, persons present, specialty, qualification of the physicians) was evaluated. Furthermore, the medical documentations were reviewed regarding compliance to the forensic standard and court usability (extent of examination and evidence collection, description of injury morphology, use of a body scheme, photographic documentation).

Data were evaluated using the “IBM SPSS Statistics” software package (version 23). Frequencies, mean values, and medians were determined for descriptive analysis. To visualize differences in the injury documentation and evidence collection between the two groups, variables that affected these characteristics were evaluated by determining chi-square test. A *p*-value of 0.05 was considered as statistically significant.

## Results

### Study group

In the case group (*n* = 132), 96.2% were injured (63.8% male, 36.2% female) and 3.8% were male defendants. The average age was 27.5 years (0–84 years).

In the control group (*n* = 132), 81.8% of those examined were injured (54.6% male, 45.4% female); in 18.2% of the cases, male defendants were investigated. The average age was 29.1 years (0–88 years).

### Circumstances of the examination

**Fig. 1:**
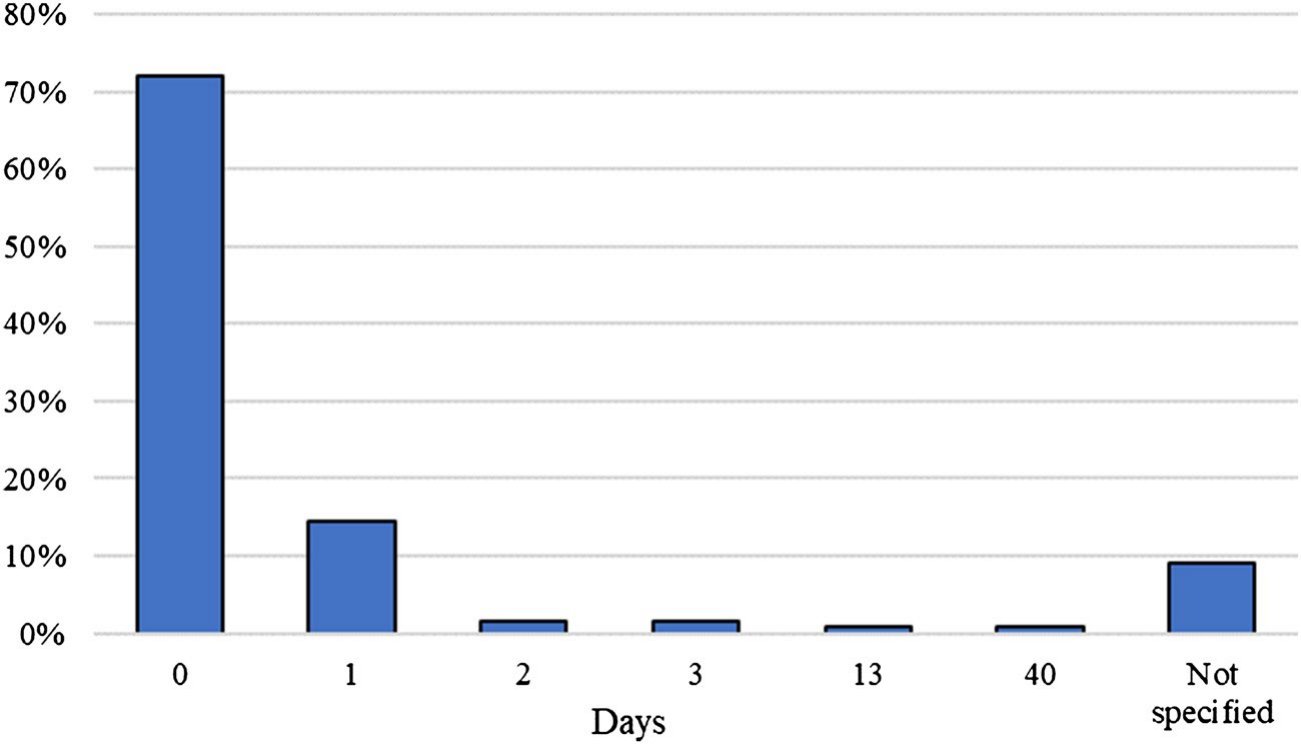
Days between incident and examination in case group

**Fig. 2:**
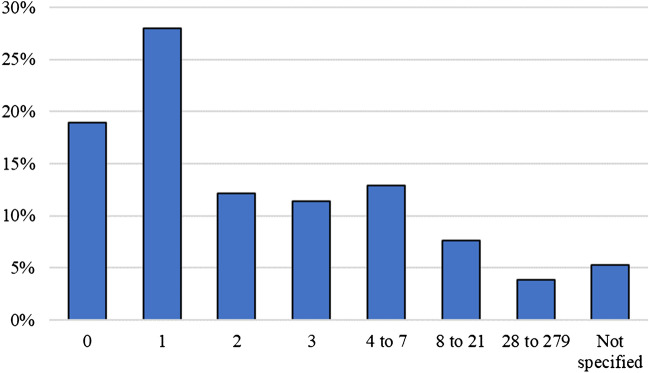
Days between incident and examination in control group

In the case group, examinations were most frequently performed on the day of the incident or the following day (95.0%, *n* = 114) (Fig. 1). In the control group, 49.0% (*n* = 62) of examinations occurred on the day of the incident or the following day, but examinations were frequently performed longer after the incident (Fig. 2).

Other circumstances of the investigations in the case and control groups are summarized in Table 1. In the case group, there were significantly more examinations during duty hours (40.9%, *n* = 54) than during regular working hours (6.1%, *n* = 8). However, the examination time could not be traced in almost half of the cases. The exact duty time models in the individual clinics were not known. Therefore, regular working hours were determined as Monday through Friday from 7:00 a.m. to 5:00 p.m. Accordingly, duty hours were determined to be Monday through Friday from 5:01 p.m. to 6:59 a.m., as well as Saturdays and Sundays. Most of the examinations in the case group were conducted in hospitals (90.9%, *n* = 120). Rarely, an established doctor’s office was visited for examination (5.3%, *n* = 7), and one person (0.8%) was examined at the police headquarters. The examinations were rarely accompanied by the police (6.1%, *n* = 8). In the case group, the examining medical department was frequently not documented (36.4%, *n* = 48). 40.9% (*n* = 54) of the examinations took place in surgery. The remaining examinations were distributed among the specialties of gynecology (3.8%, *n* = 5), pediatrics (14.4%, *n* = 19), general medicine (3.0%, *n* = 4), and other (neurology, neurosurgery) (1.5%, *n* = 2). Information on the qualifications of the examiners in the case group was missing in 77.3% (*n* = 102); of the remaining examinations, 21.1% (*n* = 28) were performed by specialists and 1.5% (*n* = 2) by residents.

**Table 1:**
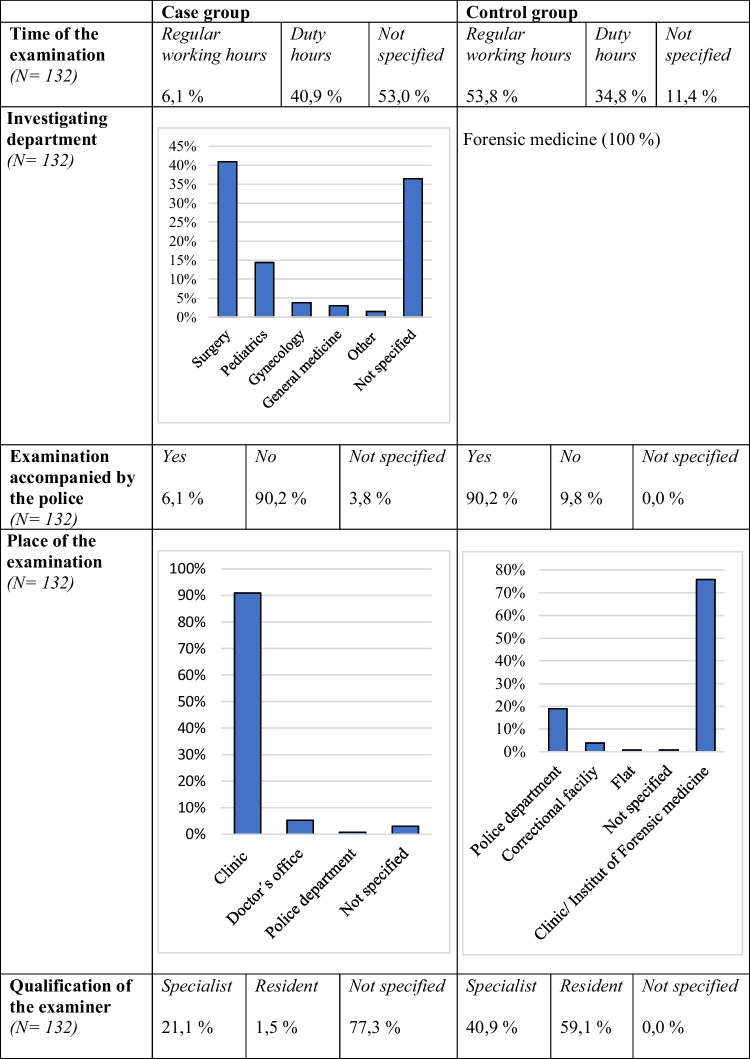
Circumstances of the examinations

The examinations in the control group were performed during regular working hours in 53.8% of the cases (*n* = 71), and 34.8% (*n* = 46) took place during duty hours. Regular working hours in forensic medicine are Monday through Thursday, 8:00 a.m. to 4:00 p.m., and Friday, 8:00 a.m. to 1:00 p.m. The remaining times are duty hours. In accordance with the study design, examinations in the control group were performed exclusively by forensic medicine. The examinations were performed in clinics including the Institute of Forensic Medicine (75.8%, *n* = 100), police department (18.9%, *n* = 25), and correctional facility (3.8%, *n* = 5), and one examination was conducted in the flat of the examined person (0.8%). Police was present in 90.2% (*n* = 119) of the examinations. It was found that 40.9% (*n* = 54) of the examinations were performed by specialists and 59.1% (*n* = 78) by residents.

### Type of violence and findings

In the case and in the control group, blunt force trauma was present in more than half of the cases (case group 58.0% vs. control group 54.5%), followed by sharp force trauma (case group 24.6% vs. control group 21.4%). Sexual violence (case group 8.4% vs. control group 12.3%) and violence against the neck (case group 4.2% vs. control group 8.5%) were more common in the control group than in the case group. Shaken baby syndrome (case group 2.8% vs. control group 1.3%) and gunshot (case group 2.1% vs. control group 2.0%) were rare violent events in both groups. In both groups, the external findings were dominated by hematomas, abrasions, skin redness, and stab/cut wounds. Internal findings occurred more frequently in the case group than in the control group (case group 25.0%, control group 17.4%).

### Influence by drugs and toxicological examinations

In the case group, impairment due to alcohol, drugs, or medication was reported in 30.3% (*n* = 40) of the examinations. The data were distributed between alcohol (90.0%, *n* = 36) and unspecified drugs (10.0%, *n* = 4). Collection of blood and urine samples was documented in 37.0% (*n* = 50) of the examinations in the case group. Considering only the cases in which influence by alcohol or drugs had been indicated at the time of the examination, samples were taken in 20 cases (55.0%), no samples were taken in 9 cases (22.5%), and in 9 other cases (22.5%), no information was available in this regard (Table 2).

**Table 2:** Compliance with forensic standards in the case group and the control group

	Case group			Control group			
	Total	Yes	Not specified	Total	Yes	Not specified	*p*-value
Full-body examination	*N* = 132	37.9%	0.8%	*N* = 132	93.9%	0.0%	*p* = 0.000
Examination of the anogenital region (if findings could have been expected)	*N* = 12	83.3%	8.3%	*N* = 19	100.0%	0.0%	
Examination of hidden body parts	*N* = 132	9.8%	1.5%	*N* = 132	94.7%	0.0%	*p* = 0.000
Photo documentation complete	*N* = 93	20.4%	17.2%	*N* = 117	94.4%	0.0%	*p* = 0.000
Photos with scale	*N* = 93	23.7%	17.2%	*N* = 117	90.6%	0.0%	*p* = 0.000
Overview picture	*N* = 93	43.0%	17.2%	*N* = 117	88.0%	0.0%	*p* = 0.000
Detail view	*N* = 93	71.0%	15.1%	*N* = 117	99.1%	0.0%	*p* = 0.000
Regular sharpness of the photos	*N* = 93	23.7%	36.6%	*N* = 117	94.9%	0.0%	*p* = 0.000
Regular exposure of the photos	*N* = 93	22.6%	46.2%	*N* = 117	99.1%	0.0%	*p* = 0.000
Discreet photos	*N* = 93	80.6%	17.2%	*N* = 117	99.1%	0.0%	*p* = 0.336
Body scheme used	*N* = 132	8.3%	0.0%	*N* = 132	65.9%	0.0%	*p* = 0.000
Injuries described in words	*N* = 132	99.2%	0.0%	*N* = 132	100.0%	0.0%	*p* = 0.316
Description complete	*N* = 132	71.8%	0.0%	*N* = 132	96.2%	0.0%	*p* = 0.000
Description type of injury	*N* = 132	96.2%	0.0%	*N* = 132	97.7%	0.0%	*p* = 0.466
Description size of the injury	*N* = 132	41.2%	0.0%	*N* = 132	96.2%	0.0%	*p* = 0.000
Description color of the injury	*N* = 132	9.9%	0.0%	*N* = 132	97.0%	0.0%	*p* = 0.000
Description texture of the injury	*N* = 132	35.1%	0.0%	*N* = 132	97.0%	0.0%	*p* = 0.000
Description form of injury	*N* = 132	16.8%	0.0%	*N* = 132	97.0%	0.0%	*p* = 0.000
Interpretive description	*N* = 132	12.9%	0.0%	*N* = 132	0.0%	0.0%	*p* = 0.000
Technically incorrect description of injury	*N* = 132	36.4%	0.0%	*N* = 132	0.0%	0.0%	*p* = 0.000
Generalized description of the injury	*N* = 1320, 316	27.3%	0.0%	*N* = 132	1.5%	0.0%	*p* = 0.000
Collection of blood/urine/hair samples (if substance influence was reported)	*N* = 40	55.0%	22.5%	*N* = 48	75.0%	4.2%	
Forensics performed (if traces on the body could have been expected)	*N* = 9	88.9%	0.0%	*N* = 16	100.0%	0.0%	

When toxicological examinations were carried out after being commissioned by the investigating authorities, cannabis and benzodiazepines were detected in 16.7% (*n* = 2) each and cocaine in 8.3% (*n* = 1). Blood alcohol analysis was positive in 15.2% (*n* = 20); the concentrations ranged from 0.48 to 2.84‰ (M = 1.43, med = 1.35, SD = 0.74).

In the control group, impairment due to alcohol/drugs or medication was reported in 36.4% (*n* = 48) of the examinations. The data were distributed between alcohol (90.2%, *n* = 46) and unspecified drugs (9.8%, *n* = 5). Collection of blood and urine samples was documented in the control group in 37.9% (*n* = 50) of the examinations. Considering only cases in which a substance influence was indicated at the time of the examination (*n* = 48), samples were taken in 75.0% of these cases (*n* = 36). In 20.8% (*n* = 10), no blood or urine samples were taken, and in 4.2% (*n* = 2), this was not evident from the files (Table 2).

The toxicological examinations carried out on behalf of the investigating authorities revealed cannabis in 22.2% (*n* = 4), amphetamine in 11.1% (*n* = 2), and cocaine, opiates, and antidepressants in 5.6% (*n* = 1) each. Blood alcohol analysis was positive in 15.9% (*n* = 21); the concentrations ranged from 0.1 to 3.99‰ (M = 1.28, med = 1.29, SD = 0.85).

### Documentation of injuries and trace evidence

The medical documentations were examined for compliance with the forensic standard required in the examination of victims of violence and corresponding court proceedings. For this purpose, it was evaluated whether a full-body examination including the concealed parts of the body (e.g., oral mucosa and ocular conjunctiva) as well as, in case of corresponding indications, an anogenital examination, securing of evidence, and taking of blood and urine samples was performed. For injury documentation, it was evaluated whether there was written documentation of findings with a description of injury morphology (type, size, color, shape, and nature of injuries) and whether care was taken to provide a purely descriptive rather than interpretive description. In addition, we evaluated whether complete photographic documentation was prepared in compliance with the forensic standard (e.g., scale, overview, and detail) and whether a body diagram was used.

The analysis revealed statistically significant differences between the studied groups in almost all variables (chi-square test), except for making a written documentation of injuries, documenting the type of injury, and the discreteness of the photo documentation (Table 2).

In the case group, a whole-body examination was performed in only 37.9% (*n* = 50) of cases, and the concealed parts of the body were examined in only 9.8% (*n* = 13) of clinical examinations. If a whole-body examination or an examination of the concealed body parts was not performed in the control group, there was a comprehensible reason in all cases (refused, not possible, in accordance with the police order). In the case group, no justification could be obtained from the files in any case.

In the present 12 cases of sexual violence in the study group, an examination of the anogenital region was performed in only 10 cases (83.3%). In the control group, in all 19 cases of sexual violence, an examination of the anogenital region was performed, either by forensic medicine (94.7%, *n* = 18) or, in one case (5.3%), clinically beforehand.

If there were corresponding indications of traces on the body of the examined persons, evidence recovery was performed in 88.9% (*n* = 9) of the examinations in the case group and in all (*n* = 16) of the examinations in the control group.

Collection of blood, urine, or hair samples was taken in 55.0% (*n* = 40) of examinations in the case group and 75.0% (*n* = 48) of examinations in the control group for evidence of (a history of) substance impairment.

Injuries were described in both groups in all examinations, unless the person examined had no findings (case group 99.2%, *n* = 130; control group 100.0%, *n* = 131). The case group very often lacked information on the size (58.8%, *n* = 77), color (90.1%, *n* = 118), shape (83.2%, *n* = 109), and texture (64.9%, *n* = 85) of the injuries. Furthermore, in the case group, injuries were described in a technically incorrect, generalizing, or interpretive manner in approximately one in three examinations. Terms such as “bruise mark,” “impact-related upper lip hematoma,” and “multiple hematomas of different ages” were used.

When information on photo documentation was available in the case group, only in 20.4% (*n* = 19) of the cases all described injuries were depicted. In the control group, the photo documentation was complete in 94.4%. In addition, compared with the control group, the photos in the case group were very often taken without scale (59.1%, *n* = 55), lacked overview (39.8%, *n* = 37), and detail shots (13.9%, *n* = 13) and were of limited shooting quality (insufficient sharpness (39.7%, *n* = 37) and exposure (31.2%, *n* = 29)). In the case group, there were special reasons when a scale was not used, for example, when the injury was localized in a body region such as the oral cavity or anogenital region.

In the case group, a body scheme was used only very rarely (8.3%, *n* = 11), but it was striking that a body scheme was frequently not used in the control group either (34.1%, *n* = 45).

## Discussion

The issue of violence is becoming increasingly important to public health, especially as the causes and consequences of violence are better understood and the role of the health care system becomes more defined [12]. Medical knowledge not only serves to treat pathological changes in the body but also fulfills social functions. In the case of persons who have experienced physical or sexual violence, it helps in particular to clarify the criminal background and to convict the perpetrators [13].

This study demonstrates statistically significant deficiencies in the quality of medical examinations of victims of violence by clinicians concerning the scope of the examination, court usable documentation of injuries, and evidence collection.

Only a few publications deal with partial aspects of the usability of medical reports in criminal proceedings [14–19]; comprehensive analyses have not been found in the international scientific community.

The study population was comparable in both groups and consisted predominantly of aggrieved persons, who were more often male than female. The investigated defendants were exclusively men. The examined persons covered all ages in both groups, and the average age was 27.5 and 29.1 years, respectively. The data are comparable to other studies in which, when non-domestic violence is considered, approximately 73–82% of the victims are male and the average age is at about 30 years [15, 17, 20]. The control group was matched to the case group based on the type of violence involved, to evaluate case circumstances that were as comparable as possible as a basis for the studies. Most examinations took place after blunt and sharp force trauma, which is comparable to other studies [15, 20]. The range of external injuries was roughly comparable in both groups, with stab/cut wounds documented more frequently in the case group. Internal injuries were also more common in the case group, which fits by the urgent medical treatment required in most of these cases. Often, the injuries had already been clinically documented and treated (e.g., cut and stab wounds), so investigating authorities did not order an additional forensic medical examination of the person but rather an expert opinion based on the medical documentation.

In both groups, examinations most frequently took place on the day of the incident or the following day, although it was not uncommon that especially the forensic examinations also took place later. The examinations in the case group very often took place in surgical departments, which is comparable to the literature [20], but in 36%, it was not possible to determine from the medical documentations in which department the examination was performed. In the case group, the examination frequently took place during duty hours, in the control group more frequently within regular working hours. In the case group, however, the examination time could not be traced in almost half of the cases. The qualifications of the examining physicians could also not be determined from the documentation in a large proportion of the non-legal medical examinations. However, an indispensable prerequisite for a record that can be used in court is the documentation of the date, time, and place of the examination as well as the examining person [21]. Once again, the principle of “in dubio pro reo” applies if it cannot be determined with certainty that the medical documentation of findings took place chronologically after the assault. In addition, plausibility checks of data on the time of occurrence of injuries are not possible. As a limitation, it must be noted that the examiners, the examining departments, and the time of examination may not had been included in the forensic expert report, and the original documents had already been returned to the courts.

The forensic medical examinations very often took place in the company of the police. In the case group, the police were rarely present. This could be due to the fact that the examinations in these cases were more often conducted without prior filing a criminal complaint. As a limitation of the study, it has to be mentioned that in the clinical cases, the examinations have another purpose. The medical treatment (diagnostic and therapeutic issues) and not the court usable documentation is in the foreground for the treating physicians. In addition, in clinical practice, the large number of patients and associated time pressure makes it difficult to recognize and accurately document injuries caused by violence. Nevertheless, in the vast majority of cases, the injury pattern or the information provided indicates a previous physical or sexual assault. The presence of the police could have a positive influence on the quality of medical examination and the securing of findings, as the forensic aspects are thus made more aware to the doctors. However, the majority of victims of violence, especially women who have suffered domestic violence, do not talk about the violence they have experienced and do not file criminal charges immediately after the crime [22]. Finally, it should be particularly noted that the Istanbul Convention provides for the confidential collection of evidence even without prior criminal charges.

In cases not examined by forensic medicine, a full-body examination was not performed in over half of these cases. Even more rarely, the hidden parts of the body were inspected. An examination of the entire body for injuries is relevant from a forensic point of view, since there may be relevant findings even apart from described symptoms (e.g., pain) and injuries requiring medical treatment [9, 23]. Significant findings can often be delineated on non-obvious body sites such as the eyelids and conjunctiva, oral mucosa, and posterior ear regions following violent offenses. After a compressive violence against the neck, petechiae can result in these areas, which allow a conclusion to be drawn about the dangerousness of the attack and are therefore also significant for the legal evaluation. Following blunt force trauma to the oral region (e.g., covering of the airway and beatings), findings are often demarcated in the oral mucosa alone and can be easily overlooked. In cases of sexual violence involving manipulation or penetration in the genital or anal area, anogenital examination is appropriate depending on the temporal circumstances but should always be offered in acute cases. Ultimately, only a complete examination can be meaningfully interpreted regarding the course of the crime.

The injuries were described in writing in both groups in all cases, in which injuries were present. However, in the case group, compared to the control group, the description was incomplete in some cases and information on the size, color, shape, and texture of the injuries was missing very frequently. In the case group, generalizing, interpretive, and technically incorrect descriptions of injuries were found in approximately one in three examinations. An exact and purely objective description of findings is of great importance for a later reconstruction of the crime by experts in criminal proceedings (e.g., forensic physicians). Besides, it belongs to the exclusive competence of the courts to answer matters of law, in particular the commitment of a crime. Generalized descriptions such as “multiple older hematomas on the body” or technically incorrect descriptions of types of injuries such as “bruising marks” do not allow reliable conclusions to be drawn about the violence involved.

Medicolegal literature calls for photographs with scale and/or drawings of injury patterns to document injuries [9, 23]. A body chart was used in the case group only in individual cases. However, it was striking that even in the control group, a body chart was used in only 65.9% of the examinations and thus not as standard. It is therefore questionable what added value the body chart still has in the age of digital photography. At least in the case of multiple injuries, documentation in a body chart is considered helpful [24].

In the forensic context, at least two photographs are to be taken for each injury, one overview and one detail photograph. In addition, the photos must be adequately exposed and have sufficient sharpness [9, 24, 25]. Photo documentation was incomplete in 62.4% of the case group. The photos taken in the case group were of significantly limited quality compared to the control group; as often no scale was used, there was limited sharpness or exposure, and overview photos were mostly missing. Also, the persons were sometimes portrayed indiscreetly (e.g., full-body shot of completely undressed person), which could result in victimization of the persons affected and rejection of further investigations.

In the few cases where traces on the body were to be expected, swabs were secured in all forensic examinations; in the case group, the securing of traces was absent in one case. In this case, unlike the other investigations, the police were not present. In Germany, the police generally hands over the necessary materials for evidence recovery, so there are clear work orders for the examining physicians in these cases. The presence of the police can have a positive influence on the completeness of the examinations. In cases without criminal charges, the problem in clinics is that the necessary materials to preserve evidence are often not available.

According to literature, persons affected by violence are under the influence of alcohol or drugs in approximately 41 to 52% [26–28]. In the present study, a substance impairment was suspected in 30.3% (case group) and 36.4% (control group) of the examinations based on the previous history. Collection of blood, urine, or hair samples was not obtained in more than half of the clinical examinations and three quarters of the forensic medical examinations despite appropriate evidence of substance impairment in the history. An influencing factor in these cases could be that the assay of blood, urine, or hair samples was not explicitly commissioned by the police, which according to forensic experience occasionally occurs. If the examination initially took place without a criminal complaint being filed, there is also the problem of forensic preservation of the samples in the clinical examinations. If there is no cooperation with an institute of forensic medicine, storage options that are court approved and only accessible to authorized persons are usually not possible.

In the last decade, several guidelines have been published by professional societies that address the forensically relevant aspects in medical examinations of victims of violence [9, 21, 23, 29, 30]. However, considering the results of the present study, these do not seem to be effective or to receive sufficient attention on a broad scale. The quality deficiencies identified in this study can lead to significant limitations in law enforcement and retraumatization of the individuals involved.

## Conclusion

To improve the situation for persons affected by violence and in pending criminal proceedings, a detailed integration of contents of clinical forensic medicine in medical studies is necessary.

Moreover, nationwide structures must be created to provide access to sound clinical forensic care. For the establishment of such a network, which is also required by the Istanbul Convention, a close connection to forensic medicine is indispensable to guarantee the necessary forensic standard in the examinations for subsequent criminal proceedings.

The use of standardized examination forms and materials (e.g., examination kits) for both—justice-initiated and low-threshold cases—regular training of medical staff on forensic quality characteristics and telemedicine approaches can improve the care situation for victims of violence regarding criminal prosecution.

## Data Availability

The datasets generated during and/or analyzed during the current study are available from the corresponding author on reasonable request.
